# The Plague of 1871 in Buenos Ayres

**Published:** 1872-04

**Authors:** 


*The Plague of* 1871 *in Buenos Ayres.*

A pestilence has just swept over Buenos Ayres, which, if not equal in fatality to some of the epidemics in Europe during the seventeenth century, is almost without a parallel in modern times. Two accounts of this great disaster have been published, one by the Rev. T. E. Ash, B.A., Chaplain of British Legation at Buenos Ayres; the other—more strictly medical—by William N. Hiron, M.R.C.S., and it has occurred to us that some useful sanitary lessons might be gathered from the graphic and instructive narratives which these gentlemen have given.

Buenos Ayres, the capital of La Plata, the city of "good air," is a town of some 180,000 inhabitants, situated on the banks of the river Platte. Until recently it has borne a high reputation for health; "as far as natural climate, air and soil of the country are concerned, unquestionably the healthiest place in the world," is Mr. Ash's description. Alas, that man, by indifference and neglect, should have made it what it is! By what accident or providence it has escaped so long and avoided a catastrophe is one of those mysteries that we cannot attempt to solve.

Imagine a city with narrow streets, crowded houses, and, in parts, a density of population almost beyond belief; where there is no provision for drainage beyond the cess-pool or old well; no water-supply save that from the river—a river so poisoned by filth, that "dead fish covered the roadstead as high as Palermo." Add to this a sluggish inlet, into which the Riachuelo (our little Ganges, as it was aptly termed) poured a reeking mass of *debris* from the slaughter-houses, which may be better imagined than described; "the saladeros continued working, and the river at Baraccas literally ran blood; the smell in December had been so horribly nauseous that, in various parts of the town, ladies and people of weak constitution were seized with vomiting when the wind blew from the south."

Newly-made roads had been filled in with offal and refuse from the scavengers' carts before being macadamized, and these gave forth an almost intolerable stench after every shower of rain.

Cesspools have been mentioned as universal, and as many as fifteen or sixteen old wells would be found in clearing the site for a house. These naturally became the receptacles of filth and of water, which it was prohibited to throw in the streets. The soil, therefore, in spite of a dry winter and spring, was saturated with moisture, and a summer sun of unprecedented power brought about the natural results. The summer months in Buenos Ayres are December, January, and February. The plague, therefore, which lasted from January to May, took place in the true epidemic season, the latter half of summer and autumn. "The city was fermenting and steaming; so noxious and deadly were the vapors that rose from the ground, that wherever it was opened nausea and sickness followed." In the expressive words of Mr. Ash, "the air was foul and sickening, the water was corrupted, the earth was reeking with abomination. The plague came, and it found the place ripe for destruction."

At the close of 1870, Asuncion, the capital of Paraguay, was attacked, and soon afterwards Corrientes, a town on the direct line of communication between Asuncion and Buenos Ayres. With the new year of 1871 it became manifest that people were dying fast in one of the low quarters of Buenos Ayres: "it was cautiously whispered, 'we have yellow fever amongst us,' but Dr. Golfarini and others hastened indignantly to contradict the rumor, and comfort the public mind. It was only the fall of the leaf, etc."

No precautions were taken, no sanitary *cordon* drawn round the infected quarter. In February came the Carnival, with its crowded theatres and noisy festivities, and the mortality was doubled. The municipality now became alarmed, and precautions were taken, but whitewash and offal carts, and sprinkling of tar, were inadequate to cope with evils now grown so gigantic. In March, the deaths reached 350 in the day, and all parts of the city were involved. Physicians recommended those who could to leave the city, and in the general *sauve qui peut* no less than 100,000 fled from their homes. All honor to those who, in that fearful time, were true to duty. "The few English doctors," we read, "stood their ground manfully," and were all at one time or another struck down, but, fortunately, recovered. The Irish nuns, the French sisters of charity, and the clergy of English, Scotch, Irish and American congregations, bore an heroic part, where friends and neighbors had fled in wild dismay. "It was like a gleam of sunshine to see the French and Irish sisters noiselessly moving about on their mission of mercy, soothing the last hour of many, and sometimes rescuing others from the jaws of death, who had been forsaken by friends and kindred."

Bakers and printers suffered most severely; no less than sixteen newspaper carriers died. Women and children almost escaped, and grave-diggers, though overworked, bore a charmed life; "there were 360 employed, and yet not one died of the fever."

The difficulties of interment increased with the mortality, and many bodies lay for days unburied. Men, supposed dead, broke from their flimsy coffins on the way to the grave. Buenos Ayres was as a city of the dead; business was at a standstill. "At night the silence was rarely broken but by the hollow sound of vehicles taking off the dead, or the tinkling of a little bell, as the blessed sacrament was conveyed to the dying."

Instances of heroic devotion even unto death were numerous. The cases of one merchant and his wife, stricken down together, were "so malignant that six nurses in succession died while attending on them, and finally a young lady, of good family and education, volunteered for the post of danger, and also fell a victim." The doctors had toiled nobly. "Of seventy who remained on duty, about half sickened, and fifteen died."—(Hiron.) The mortality for the month was 11,000.

By the middle of April all the city offices had closed except four, and twenty days' vacation was ordered by the Government; but more than 22,000 persons had been buried in one cemetery during the past three months.

During the latter part of April and May a marked and rapid improvement took place, until, with the advent of winter, the plague was finally extinguished. Of the cases imported to the country districts none spread, and in other places, such as Parana, Rosario, and Monte Video, immunity was secured by a strict and vigilant quarantine. Assistance readily poured in from various quarters. Monte Video sent up 10,000*l.* for the poor; Brazil, Chili, and Banda Oriental were not behind, and in May last large sums of money, together with disinfectants and papers of advice from the sanitary authorities, were promptly forwarded from England to the desolated city.

More recently Dr. Scrivener has been sent over to Europe by the Government of Buenos Ayres to inform himself on all matters of hygiene likely to be of use in averting or combating any future outbreak of the disease. The causes of a mortality so disastrous would not seem far to seek: decomposition of animal and vegetable matters of the vilest description, under an almost tropical sun, poisoned drinking supplies, densely crowded and filthy fever nests, invited the outburst of epidemic disease, and fed it to the last; whilst an imperfect sanitary administration could but look on with dismay.

The losses from the plague were variously estimated at from 20,000 to 26,000, and at one time there remained but 40,000 people in the town, of whom 7,000 were sick, with a daily mortality of 600 or 700.

Such was the plague of the year just ended in Buenos Ayres. It will be well if a lesson has been learned, and a desire for sanitary improvements impressed deeply on the people.—*Food Journal.*

